# Metabolomic database annotations *via *query of elemental compositions: Mass accuracy is insufficient even at less than 1 ppm

**DOI:** 10.1186/1471-2105-7-234

**Published:** 2006-04-28

**Authors:** Tobias Kind, Oliver Fiehn

**Affiliations:** 1 University of California Davis, Genome Center, 451 E. Health Sci Dr., Davis, CA 95616, USA

## Abstract

**Background:**

Metabolomic studies are targeted at identifying and quantifying all metabolites in a given biological context. Among the tools used for metabolomic research, mass spectrometry is one of the most powerful tools. However, metabolomics by mass spectrometry always reveals a high number of unknown compounds which complicate in depth mechanistic or biochemical understanding. In principle, mass spectrometry can be utilized within strategies of *de novo *structure elucidation of small molecules, starting with the computation of the elemental composition of an unknown metabolite using accurate masses with errors <5 ppm (parts per million). However even with very high mass accuracy (<1 ppm) many chemically possible formulae are obtained in higher mass regions. In automatic routines an additional orthogonal filter therefore needs to be applied in order to reduce the number of potential elemental compositions. This report demonstrates the necessity of isotope abundance information by mathematical confirmation of the concept.

**Results:**

High mass accuracy (<1 ppm) alone is not enough to exclude enough candidates with complex elemental compositions (C, H, N, S, O, P, and potentially F, Cl, Br and Si). Use of isotopic abundance patterns as a single further constraint removes >95% of false candidates. This orthogonal filter can condense several thousand candidates down to only a small number of molecular formulas. Example calculations for 10, 5, 3, 1 and 0.1 ppm mass accuracy are given. Corresponding software scripts can be downloaded from . A comparison of eight chemical databases revealed that PubChem and the Dictionary of Natural Products can be recommended for automatic queries using molecular formulae.

**Conclusion:**

More than 1.6 million molecular formulae in the range 0–500 Da were generated in an exhaustive manner under strict observation of mathematical and chemical rules. Assuming that ion species are fully resolved (either by chromatography or by high resolution mass spectrometry), we conclude that a mass spectrometer capable of 3 ppm mass accuracy and 2% error for isotopic abundance patterns outperforms mass spectrometers with less than 1 ppm mass accuracy or even hypothetical mass spectrometers with 0.1 ppm mass accuracy that do not include isotope information in the calculation of molecular formulae.

## Background

Metabolomics seeks to identify and quantify all metabolites in a given biological context [1]. In this respect its aim is different from metabolic fingerprinting or metabonomic approaches which utilize high dimensional unannotated variables and multivariate statistics to find biomarkers that may or may not be structurally identified in subsequent steps. Therefore, an important task in metabolomics is to identify or structurally annotate compounds in a high throughput manner. Mass spectrometry is one of the most powerful tools for unbiased analysis of small molecules in life sciences. Hundreds to thousands of metabolites can be detected when suitable sample preparation methods [2] and mass spectrometric techniques are used [3]. However, most of the metabolites in complex biological materials like plant tissues are non-annotated, unidentified metabolites [5] due to the lack of experimental databases and the chemical complexity and changing nature of an organism's metabolome. Metabolites cannot be sequenced like proteins or polynucleotides. Instead, each individual compound needs to undergo structural elucidation, starting from the elemental composition. In addition to detection and quantification of metabolites, mass spectra can further be exploited for structural elucidation of compounds [4].

**Figure 1:**
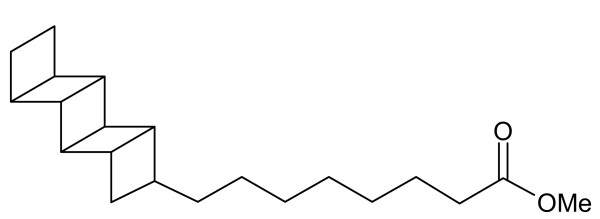
**Nature is known to synthesize "fancy" compounds**. A natural occurring ladderane produced by the anammox bacterium "Candidatus Brocadia anammoxidans"

In order to reduce the number of *de novo *elucidations for metabolomic studies, a reasonable strategy could start with tentatively annotating metabolomic mass spectra with a list of compounds that match the elemental composition of small molecules found in publicly available databases. For numerical reasons the list of potential metabolic candidates will vary with the size and the quality of the queried database, but in principle, even structures with uncommon chemical conformations like ladderanes [6] (Figure 1) cannot be excluded *a priori*. The list of tentative annotations could be further confined in subsequent steps by including additional physicochemical or biological information such as matching predicted versus determined MS/MS fragmentation patterns [7,8] or likelihood assessments from exploiting genomic knowledge about an organisms' biochemical pathways. However, without reference standards or complementary structural data (e.g. garnered by 2D nuclear magnetic resonance [21], a certain level of ambiguity will remain in purely mass spectrometric approaches due to the combinatorial explosion. It is important to note that mass spectrometry alone can not distinguish between stereoisomers.

The mass of chemical elements is based on the conventional scale that defines carbon C = 12.000 u. Chemical elements are comprised of a different number of neutrons, protons and electrons, so that the combined mass for each element (other than ^12^C) is a rational (non-integer) number: ^1^H = 1.007825 u, ^14^N = 14.003070 u, ^16^O = 15.994910 u [9]. Consequently, for any given metabolite, the accurate mass deviates from the nominal mass. This feature can be exploited for recursively calculating the elemental composition from an unknown metabolite mass spectrum in the ranges of the measurement error. Mass spectrometers today can measure mass/charge ratios with high (<5 ppm error; *parts per million*) or very high mass accuracy (<1 ppm) [11] and can be purchased with implemented software algorithms that derive a list of possible elemental compositions from the measured monoisotopic mass. Using the accurate mass one can either solve the diophantine equation [15] or one can use a brute force approach [16] and can calculate all possible elemental combinations in a certain range.

Another important prerequisite for this approach is not only accurate mass measurement but also a high resolving power of the mass spectrometer. As the output of a mass spectrum is represented as a Gaussian or Lorentzian like peak shape, very near peaks can overlap on devices with low resolving power. Resolving power (*m/Δm*) at a certain m/z value can be calculated at full-width half-height maximum (FWHM) of the peak. Quadrupole mass spectrometers usually can reach 3000 [11], TOF analyzers up to 10,000 and Fourier Transform Ion Cyclotron Resonance (FT-ICR) mass spectrometers can have a resolving power up to 1,000,000 or larger [35]. Isobaric masses (for example C_37_H_31_N_8_P_7_S_3 _MW = 899.999692 and C_20_H_43_N_2_O_19_P_3_S_6 _MW= 899.999678) can not be resolved by mass spectrometry only. In this case chromatography helps to separate these overlapping components.

For the case of peptides it was claimed that accurate mass measurements of 1 ppm error would be sufficient to derive a single solution in most cases [38]. However this is not applicable for small molecules, because they are not only derived from combinations of certain amino acids. We demonstrate in this report that even a hypothetical instrument capable of accurate mass measurements of 0.1 ppm error would not fulfill this premise when matching against a comprehensive list of chemically possible elemental compositions.

Additional information is required that can readily be gathered from mass spectra: the abundance of natural isotopes in metabolites which refer to the percentages in which the isotopes of an element are found in natural sources on earth. The isotopic abundance pattern of a metabolite's mass spectrum can serve as a powerful additional constraint for removing wrong elemental composition candidates. Isotope ratio mass spectrometers [14] exactly determine isotope abundances, however, under combustions of the original molecule into CO2 or other gases and therefore irrelevant for the calculation of elemental compositions of unidentified metabolites. In general, the theoretical isotopic abundance pattern of a molecular formula can be calculated using different approaches [12] either solving polynomial equations or using fast Fourier transformations [13]. An isotope abundance filter can be used for any mass spectrometer which can provide very low root mean square (RMS) errors for isotopic patterns, especially if the contribution of further metabolites can be ignored by coupling compound separation to mass spectrometric detection using liquid or gas chromatography (LC/MS and GC/MS). Mass spectra may include fragmentations, rearrangements, and adducts [10]. For the sake of clarity, mathematical and chemical considerations reported here are constrained to pseudo-molecular ions that are completely resolved from interfering compounds, assuming the utilization of efficient chromatography or high resolution mass spectrometry [18], or a combination of both.

## Results and discussion

### Database queries of elemental compositions

Assuming that a unique elemental composition could be derived from a mass spectrum, this molecular information can be furnished for metabolite annotation in either of two distinct ways: an exhaustive computation of all chemically possible isomeric structures or a query of databases for known (bio)chemical compounds.

**Figure 2:**
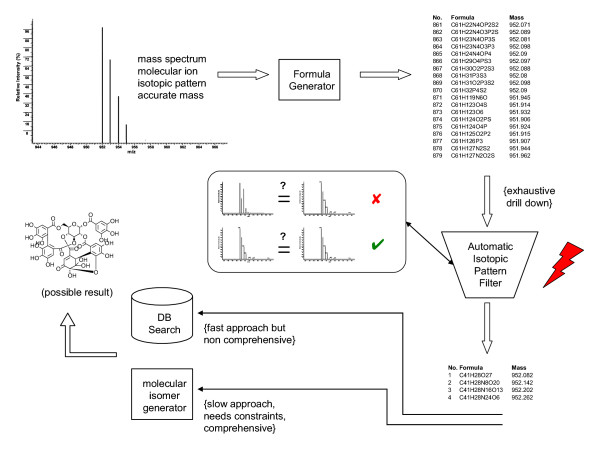
Metabolite annotation schema based on mass spectrometric calculation of elemental compositions and subsequent database queries.

Exhaustive methods (Figure 2) utilize either a deterministic approach [17] or a stochastic molecular isomer generator [27]. For a given molecular formula, several hundred to billions of isomers can be constructed, depending on the number and nature of elements given by the chemical composition. The number of molecular formulas for the eleven most common elements at 1000 u is reported to be more than 350 millions [37]. For small molecules that are analyzed by electron impact mass spectrometry, a deterministic method called MOLGEN-MS is available [20]. For high molecular weight compounds, deterministic methods are quickly challenged by computing power limits due to the combinatorial explosion of isomeric structures which may render stochastic isomer generators more promising for the future [21].

**Figure 3:**
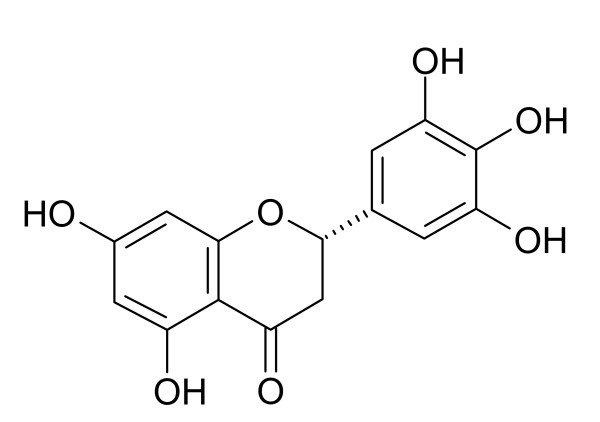
An example Pentahydroxyflavone (C_15_H_12_O_7_) taken from the KEGG database.

For automatic annotation of metabolites in metabolomic screens, it seems today more reasonable to first search against existing chemical structures or even to limit searches for known natural product databases. A randomly chosen molecular formula of C_15_H_12_O_7 _(304.0583 u) was taken as test case for query results, which should comprise structures like the naturally occurring pentahydroxyflavone (Figure 3). Seven repositories were compared for this exemplary case (Table 1): the life-science oriented PubChem database of the U.S. National Institutes of Health and its sub-DB ChemIDplus, the Kyoto ligand biochemical pathway database (KEGG), the CRC dictionary of natural products (DNP), a large compendium of organic chemical structures (Beilstein), a list of commercially available chemicals which could be used for confirming any given hit (MDL), a mass spectrum library used in GC/MS (NIST 5.0) and the complement of small molecules that have been described in the chemical and biochemical literature: the Chemical Abstracts Service (CAS) database.

A range of conclusions can be derived from this exercise (Table 1). Due to its limited size and its focus to consensus biochemical pathways, the KEGG database returned far fewer hits compared to more comprehensive repositories like CAS or DNP. It is important to note that therefore, automatic annotations of mass spectra must not be limited to KEGG searches alone. Conversely, however, any hit retrieved from KEGG queries might receive a higher likelihood of truly representing an identifiable metabolite due to the focus on (conserved) biochemical pathways represented in KEGG. In contrast to the small KEGG (Ligand) DB, the CAS database represents the largest database available for small molecules containing ~ 20 million organic chemicals. However, CAS cannot serve as suitable database for routine metabolite queries. On the one hand, CAS contains many compounds that have been artificially synthesized and reported by chemists, and thus are often unlikely to be present as natural compounds. On the other hand, the CAS SciFinder front end enables only a very limited and slow formula search, allowing queries of one formula at a time but not batches or series of queries. For these two reasons, CAS queries can be excluded from automated annotation efforts of complex metabolomic surveys; however, for identification purposes of selected unknown compounds in biomarker studies, the CAS database still provides the most comprehensive overview. It is interesting to note that DNP with only 170,000 entries retrieves 129 different isomeric structures of C_15_H_12_O_7 _(among them many stereoisomers) whereas the far larger PubChem database resulted in only 19 hits. The PubChem database is a fast growing database. At the time of search it had only 800,000 entries, now it has more than 5 millions. PubChem is a freely accessible database and includes KEGG, ChemIDplus and NCBI and several other databases and should therefore be included in automatic metabolite annotations. An in-depth molecular diversity calculation could reveal any overlap [22]. For an automated approach, the DNP database in SD file format (*.sdf) could be used whereas only semi-automatic procedures would be possible for the Beilstein database. Consequently, for identification routines of unknown metabolites starting from elemental compositions, DNP and PubChem search results should be combined.

### Calculating elemental compositions: construction of an exhaustive test data set

The input into metabolomic queries are elemental compositions which are calculated from experimental mass spectra. Often, the performance of mass spectrometers and underlying software algorithms to calculate such molecular formulae are presented on test cases. However, molecular formulae are not uniformly distributed across the mass range. In order to exhaustively test the performance and power of algorithms calculating elemental compositions, a data set containing all chemically possible molecular formulae between a molecular mass of 20 – 500 u (using the most common elements C, H, N, O, P and S) was constructed. It is wrongly assumed by researchers outside the mass spectrometry community that within that mass range, high mass accuracy calculations of <1 ppm would result in unambiguous calculation of unique elemental compositions. We therefore have applied a number of chemical constraints to reduce the number of potential elemental compositions in the exhaustive data set to only those combinations that are allowed by chemical bonding rules. Applying constraints is the most crucial step during the whole process of formula finding and structure elucidation. Consequently, we have used the molecular weight calculator MWTWIN with a variety of restrictions: the "smart H atoms" option was used to avoid the calculation of an unreasonably high number of hydrogen atoms. This excludes species like C2_6_H_2 _which are chemically possibly but not relevant for metabolomics. In extremely seldom cases this can lead to an exclusion of certain formulas with multicenter bonds (C_10_H_25_NO_4_). Secondly, metals have been excluded in our test data set because most metabolites do not contain coordinating metal atoms (although certainly a number of naturally occurring metabolites do, such as chlorophylls). However, in case trimethylsilylation was used for derivatization, search queries in GC/MS metabolite profiling data must obviously include Si which was left aside for this test data set. A third important constraint is the application of valence rules for which LEWIS and SENIOR rules were applied. These rules were found to serve as an important constraint that helped reducing an initial number of 3.5 million combinations of elemental compositions to 1.6 million for the mass range of 20–500 u (C, H, N, S O and P). Surprisingly, a number of both commercial and non-commercial formula generators are based purely on mathematical rules but do not obey the LEWIS and SENIOR chemical rules. As result, for a mass of 129.034 u species like C_9_H_5_O would be calculated by such formula generators which do not exist as natural compounds (however, which might exist as charged or radical species in the gas phase). Shortly, the LEWIS rule expects each compound to account for an even number of electrons with atoms that all obey the octet rule. SENIOR's theorem [25,26] requires three essential conditions for the existence of molecular graphs:

A) the sum of valences is an even number, or the total number of atoms having odd valences is even;

B) the sum of valences is greater than or equal to twice the maximum valence;

C) the sum of valences is greater than or equal to twice the number of atoms minus 1.

We have written scripts that include these rules in order to reduce the number of generated formulae that are exported from current commercial or non-commercial software products. The second rule was not included because it only proofs the non-existence of very small molecules like CH_2 _[26]. The current script only allows atom numbers less than 100. We have not put in a further constraint that would account for the number of and double bonds (RDBE [32]) or double bond equivalent (DBE) because for complex molecules with more than five atom types the calculation gets quite complicated. For example, nitrogen and phosphorous can have 3 or 5 valences, and sulphur atoms may have 2, 4 or 6 valences. For molecules that contain these three atoms in different valance states, no single solution for RDBE can be calculated but an RDBE range would result. An in-dept mathematical discussion of this problem can be found here [37]. Applying the LEWIS and SENIOR check is thus much more reliable and straightforward. Our current software script obeys standard valences (*ground state chemistry *[17]) in a conservative effort to produce an exhaustive number of formulas for ground state chemistry.

**Figure 4:**
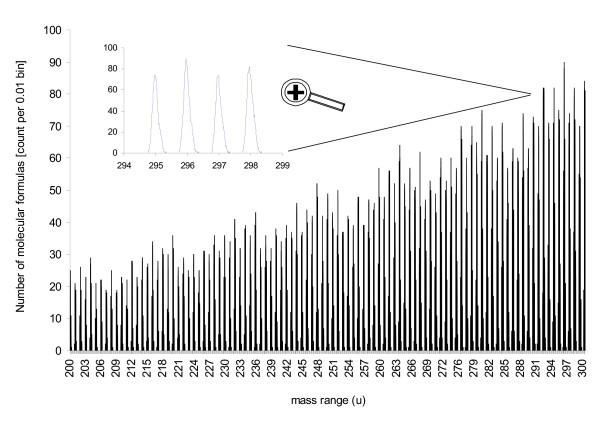
**Trend pattern histogram for mathematical possible number of molecular formulae (C, H, N, S, O and P) for the mass range 200 u-300 u**. MWTWIN with bounded search was used, LEWIS check was applied. A step size of 0.01 u was taken for counting the number of formulae.

A plot of all elemental compositions between 200–300 u is given in Figure 4. It becomes immediately clear that elemental compositions are not uniformly distributed across the mass range but recurring modalities, which are due to the dependence of elemental compositions upon the chemical constraints applied. Hence, there are large areas where not a single elemental composition exists (e.g. at 297.500 u there is no formulae within +/- 0.148 u (497 ppm mass accuracy; MWTWIN smart H option). Conversely, at maximum frequency modalities, several thousand of potential formulae are chemically allowed (e.g. around 2000 elemental compositions are retrieved between 297.74–298.34 u). Mass ranges without existing molecular formulae will shift and narrow with higher mass ranges, but peak frequencies and the characteristic pattern will remain. Consequently, the performance of mass spectrometers during elemental compositions analysis tests should be shown with masses at ± 1σ around maximum peak frequencies and not with the low number of compounds that are found at the valley of the composition distributions.

### Limits for unique molecular formula assignment

The generation of a comprehensive data set of all chemically possible molecular formulae between 20–500 u enables the prediction of the upper ppm limit for unique molecular formula assignment (see Table 2). Querying masses and formulae at peak frequency distributions from Figure 4, we have determined that this mass limit is as low as 126.000 u when the most common elements (C, H, N, S, O and P) are included and a 1 ppm mass accuracy level is assumed. With these restrictions, two chemically possible formulae are generated, C_2_H_8_O_2_P_2 _and C_3_H_2_N_4_S, both of which can be found in the CAS database and have thus indeed been reported to be existent. This level is far lower than conventionally assumed [35] and would likely be found at an even lower mass if elements like F, Cl, Br and Si were included. It is important to note that from this mass on an increasing number of formulas occur, demonstrating that <1 ppm mass accuracy alone is not sufficient for unique elemental composition assignment. Consequently, for an automatic routine, additional constraints are needed to limit the number of unique formulae from a given mass measurement.

### Accurate isotope abundance complements accurate mass measurements

Natural compounds on earth (such as metabolites from biological specimen) reflect the natural abundance of stable elemental isotopes, such as ^13^C (which is found at approx. 1.11% of the most frequent isotope ^12^C), ^18^O (0.2% of ^16^O), ^15^N (0.367 % of ^14^N), ^2^D (0.015% of ^1^H) and ^33^S and ^34 ^S (0.79 and 4.43 % of ^32^S). The actual ratios of these stable isotopes slightly differ for each element within narrow ranges [9]. Consequently, each monoisotopic pseudomolecular ion (M_0_) that is used for accurate mass determinations is always accompanied by additional isotope ions. The abundance of the isotope ions (M+1, M+2, M+3) is dependent on the actual elemental composition and can therefore serve as a powerful filter in calculating unique elemental compositions from mass spectral data. In table 3 the number of calculated elemental compositions for 150.000 to 900.000 u is given at mass accuracy levels of 10-0.1 ppm without and with additional isotope abundance information. Using conventional calculations, isotope information is not included. It is clearly seen that above approx. 200 u, mass accuracies of 3–5 ppm (an error level that is usually achieved by time-of-flight mass spectrometers, TOF [11]) lead to multiple chemically possible formulae, and to dozens of elemental compositions at masses above 400 u. It has therefore been argued to utilize the high resolving power and mass accuracy of Fourier transform ion cyclotron resonance mass spectrometers that achieve around 1 ppm average error in daily routines in unattended mode sometimes worse [18]). However, even at 1 ppm error, ambiguity of chemical formulae increases sharply above 400 u, a range in which many secondary metabolites are detected. Use of a hypothetical mass spectrometer with only 0.1 ppm error would still not result in unique solutions above 500 u, which leads to the conclusion that improving mass accuracy is not the solution for automatic assignments of elemental compositions. In contrast, applying isotope pattern recognition greatly reduces the search space for possible elemental compositions. Today, TOF mass spectrometers are available that specify 2% absolute isotope abundance accuracy at 3 ppm mass accuracy level with a resolving power of 10,000 [29]. Table 3 demonstrates that such instruments may clearly outperform the 5-fold more expensive ion cyclotron resonance mass spectrometers with respect to calculation of molecular formulae. Up to 400 u, unique solutions are achieved and between 400–800 u only 2–13 possible elemental compositions are reported. A direct comparison of the list of retrieved hits at the 3 ppm level with and without exploiting the isotope abundance information confirms that applying such an orthogonal filter above 500 u removes always more than 95% of the potential formulae. It has been argued that the chemical intuition and experience of analytical chemists would sort out unlikely chemical compositions; however, such routines cannot be implemented into query algorithms and are hard to conceive even at the 1 ppm level, when hundreds of possible hits are returned at searches between 700–900 Da, the mass range of membrane lipids. The principal idea of using a combined analysis of mass spectra and isotopic distributions is known since several decades [33,31,34]. There is a further approach called MPPSIRD (mass peak profiling from selected ion recording data) [19] in which molecular formulas with non matching ion abundances are excluded. Another approach was suggested to use isotopic pattern and "virtually" enhance the resolving power of a magnetic sector instrument from 30,000 to 90,000 or that of an FT-MS from 500,000 to 1,500,000 [24]. It has also been argued that complementary information may be garnered from mass spectral fragmentation, sometimes including accurate mass data in an intelligent basket method [28]. However such an approach is not universally applicable, and even more importantly, the interdependency of accurate mass and accurate isotope analysis for automated calculation of elemental compositions has not yet been demonstrated on a comprehensive data set of chemically possible formulae.

**Figure 5:**
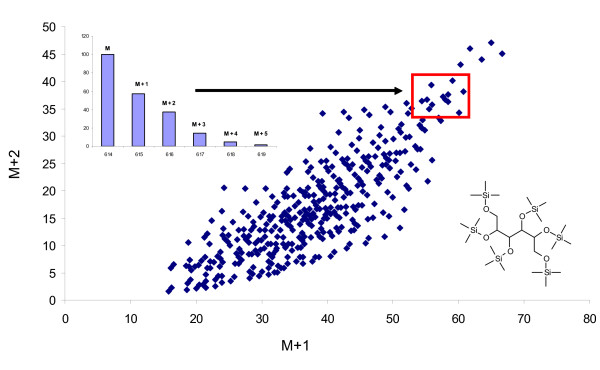
**The isotopic abundances of the M+1 and M+2 ions can be used to filter molecular formula candidates**. This example shows isotopic abundance pattern for silylated sorbitol. The red circle shows a 5% region with the correct target. All other formulae can be excluded if the mass spectrometer has a 5% error (RMS) on isotopic abundances.

A further example supports this notion of a high impact of an orthogonal isotopic pattern filter. Actual measurement data were taken from analysis of trimethylsilylated (TMS) sorbitol, which was calculated as a pseudomolecular ion with a mass/charge 615.324 u at 5 ppm error under chemical ionization using a gas chromatography – time of flight mass spectrometer (GC-TOF, [36]). In Figure 5 all 370 possible elemental compositions are plotted that are calculated from this mass including elements C, H, N, O, S, Si and P using MWTWIN with smart hydrogen option, without a restriction on the number of elements. When LEWIS and SENIOR checks were applied together with a 5% isotope abundance error, 12 result possible elemental compositions were obtained. In comparison, at 1 ppm mass accuracy still 56 formulae were calculated without orthogonal filter applied. For trimethylsilylated compounds in GC-TOF analysis, actually further constraints can be applied. After subtraction of elements counting for the trimethylsilyl group, the correct formula of the non-derivatized molecule is obtained.

## Conclusion

Based on exhaustive generation of 1.6 million molecular formulas it has been shown that high mass accuracy (1 ppm) and high resolving power alone is not sufficient for obtaining a low numbers of molecular formulas for further structure elucidation. This is especially true for molecular masses above 300 Da containing the most common elements C, H, N, S, O and P. Only an orthogonal isotopic abundance pattern filter was able to strongly reduce the number of molecular formula candidates. This of course requires mass spectrometers with a very low error for isotopic abundance distributions (RMS 1–5%). A mass spectrometer capable of 3 ppm mass accuracy but 2% isotopic pattern accuracy usually removes more than 95% of false candidates and outperforms even a (non-existing) mass spectrometer capable of 0.1 ppm mass accuracy but no isotopic pattern accuracy. Mass spectrometry producers should be enforced to provide the isotopic abundance errors in their documentation. Software producers should be enforced to use such an approach in their formula generation software for mass spectrometers.

## Methods

### Generation of molecular formulas

Exhaustive calculation of formulae from 20–500 u using C, H, N, O, S and P was performed using the Molecular Weight Calculator MWTWIN [23] on a 1.7 GHz Pentium M with 1 GByte RAM. Calculation time and data cleaning with Textpad [40] was about 24 h. As valence values and molecular masses for each of the elements are constant, the resulting patterns of these calculations are also applicable to higher mass ranges. It is feasible to calculate molecular formulas in much higher range using CHEFOEG [30]. LEWIS and SENIOR rules were checked using self-written scripts in Visual Basic which were implemented into Statistica Dataminer v7 [23] and Microsoft Excel 2003. A demo version of Molgen 3.5 [42] was downloaded and used for the calculation of the number of structural isomers of some formulae given as examples.

### Isotopic pattern filter

Isotopic pattern were calculated with a modified Mercury6 version [13]. This version takes the molecular formula as input and writes the isotopic abundances with the according masses to a log file. It can process 1 million formulas in 3 hours on a Pentium M 1.7 GHz. The resulting formulae and isotopic patterns of a single example were transferred to an MS Excel sheet where a simple matching function was implemented. Isotopic abundances are normalized to 100. The root mean square error (RMS) of the isotopic abundances is given in percent. This Excel function adds the differences between the calculated and target intensities for each of the M+1, M+2 and M+3 peaks and matches the sum of these differences against the target intensities. Furthermore an MS Excel array formula was implemented to report the number of remaining formulae when manually entering the isotope abundance accuracy in percent (according to the mass spectrometer specifications).

Mass spectrometry always reports charged species. For the correct use of the software, the neutral form of the molecule is required. In this case the charge of molecular ion can be removed and hydrogen is added or subtracted to retrieve the neutral form of the molecule (mass of proton and electron = 1.007825 u). Any other adduct must be removed in the same manner.

In table 3, isotope abundance examples were taken from individual compounds that were randomly selected from 48,000 example formulae in the range of 150–900 u, each of which had to pass LEWIS and SENIOR checks and an inclusion of C and H out of the list of C, H, N, S, O and P. Accordingly, selection of another compound for each mass example would change the single result given in the 'isotope abundance accuracy' columns, but not the overall conclusions. For all cases, the MWTWIN smart H option was applied, excluding potential formulae with a high combination of elements (e.g. C_26_H_4_) [39] that are inexistent in metabolome compositions. A complete matrix containing all results for 10, 5, 3, 1 and 0.1 ppm and 20, 10, 5, 2 and 1% isotopic abundance accuracy for 150–900 ppm can be found at .

## Authors' contributions

Both authors contributed equally to the work.

## Figures and Tables

**Table 1:** Example of a molecular formula search for C_15_H_12_O_7 _in different chemical databases. Search date: July 2007

**Database name**	**Compounds found**	**Total database entries**
Chemical Abstracts (CAS)	181	24,000,000
Beilstein Database (MDL)	166	8,000,000
Dictionary of Natural Products (DNP)	129	170,000
PubChem (NIH)	19	800,000
Available Chemicals Directory (MDL)	6	400,000
ChemIDplus (NIH)	6	370,000
KEGG (Kyoto University)	3	13,000
NIST05 (NIST mass spectral database)	2	163,000
		
MOLGEN molecular isomer generator (allowing 2 benzene groups; 1 ether group, 1 keto group; 5 hydroxy groups)	788,000	-

**Table 2:** Limits for unique formula assignment at certain levels of mass accuracy [ppm]. Above the listed mass ranges multiple formula findings cumulate. The CAS database sometimes reports D instead of H and radicals and ions as substances. Molgen was used with lowest element valence values. Formulas must contain C and H out of elements CHNSOP

		**example**	**example**	**CAS Hits**	**CAS Hits**	**MOLGEN**	**MOLGEN**
**ppm**	**mass range < [Da]**	**compound 1**	**compound 2**	**formula 1**	**formula 2**	**formula 1**	**formula 2**
0.1	185.9760	CH2N2O9	C4H11PS3	0	6	7116	1116
0.5	138.0000	C4H2N4S	C3H8O2P2	27	0	247932	353
1	126.0000	C2H8O2P2	C3H2N4S	1	27	2852	24928
2	126.0000	C2H8O2P2	C3H2N4S	1	27	2852	24928
3	126.0000	C2H8O2P2	C3H2N4S	1	27	2852	24928
4	95.9881	C3HN2P	CH4O3S	0	17	522	14
5	95.9881	C3HN2P	CH4O3S	0	17	522	14
6	95.9881	C3HN2P	CH4O3S	0	17	522	14
7	95.9881	C3HN2P	CH4O3S	0	17	522	14
8	95.9881	C3HN2P	CH4O3S	0	17	522	14
9	95.9881	C3HN2P	CH4O3S	0	17	522	14
10	93.9911	CH2O5	C2H6S2	1	22	9	5
20	77.9788	CH2O2S	CH4P2	13	20	9	4

**Table 3:** Number of possible molecular formulas at different levels of mass accuracy and the impact of isotopic abundance accuracy. A mass spectrometer capable of 3 ppm but with 2% correct isotopic pattern outperforms even a (non-existing) mass spectrometer with 0.1 ppm mass accuracy! The results are computed for randomly selected targets, so single results vary but the trend remains. LEWIS and SENIOR check was applied. Candidates with unrelated high element counts were already excluded

	without isotope abundance information					2% isotopic abundance accuracy	5% isotopic abundance accuracy
						
molecular mass [Da]	10 ppm	5 ppm	3 ppm	1 ppm	0.1 ppm	3 ppm	5 ppm
150	2	1	1	1	1	1	1
200	3	2	2	1	1	1	1
300	24	11	7	2	1	1	6
400	78	37	23	7	1	2	13
500	266	115	64	21	2	3	33
600	505	257	155	50	5	4	36
700	1046	538	321	108	10	10	97
800	1964	973	599	200	20	13	111
900	3447	1712	1045	345	32	18	196
